# Different Stratification of Physiological Factors Affecting Cerebral Perfusion Pressure in Hypoxic-Ischemic Brain Injury after Cardiac Arrest According to Visible or Non-Visible Primary Brain Injury: A Retrospective Observational Study

**DOI:** 10.3390/jcm10225385

**Published:** 2021-11-18

**Authors:** Changshin Kang, Wonjoon Jeong, Jung Soo Park, Yeonho You, Jin Hong Min, Yong Chul Cho, Hong Joon Ahn, Yong Nam In, In Ho Lee

**Affiliations:** 1 Department of Emergency Medicine, Chungnam National University Hospital, 282 Munhwa-ro, Jung-gu, Daejeon 35015, Korea; changsiny@naver.com (C.K.); gardenjun@hanmail.net (W.J.); yyo1003@naver.com (Y.Y.); boxter73@naver.com (Y.C.C.); jooniahn@daum.net (H.J.A.); 2 Department of Emergency Medicine, College of Medicine, Chungnam National University, 282 Mokdong-ro, Jung-gu, Daejeon 35015, Korea; laphir2006@naver.com (J.H.M.); ynsoft@naver.com (Y.N.I.); 3 Department of Emergency Medicine, Chungnam National University Sejong Hospital, 20 Bodeum 7-ro, Sejong 30099, Korea; 4 Department of Radiology, College of Medicine, Chungnam National University, 282 Mokdong-ro, Jung-gu, Daejeon 35015, Korea; leeinho1974@hanmail.net

**Keywords:** out-of-hospital cardiac arrest, hypoxic-ischemic brain injury, targeted temperature management, primary brain injury, secondary brain injury

## Abstract

We aimed to explore the stratification of physiological factors affecting cerebral perfusion pressure, including arterial oxygen tension, arterial carbon dioxide tension, mean arterial pressure, intracranial pressure (ICP), and blood-brain barrier (BBB) status, with respect to primary or secondary brain injury (PBI or SBI) after out-of-hospital cardiac arrest (OHCA). Among the retrospectively enrolled 97 comatose OHCA survivors undergoing post-cardiac arrest (PCA) care, 46 (47.4%) with already established PBI (high signal intensity (HSI) on diffusion-weighted imaging (DWI) had higher ICP (*p* = 0.02) and poorer BBB status (*p* < 0.01) than the non-HSI group. On subgroup analysis within the non-HSI group to exclude the confounding effect of already established PBI, 40 (78.4%) patients with good neurological outcomes had lower ICP at 24 h (11.0 vs. 16.0 mmHg, *p* < 0.01) and more stable BBB status (*p* = 0.17 in pairwise comparison) compared to those with poor neurological outcomes, despite the non-significant differences in other physiological factors. OHCA survivors with HSI on DWI showed significantly higher ICP and poorer BBB status at baseline before PCA care than those without HSI. Despite the negative DWI findings before PCA care, OHCA survivors have a cerebral penumbra at risk for potentially leading the poor neurological outcome from unsuppressed SBI, which may be associated with increased ICP and BBB permeability.

## 1. Introduction

Out-of-hospital cardiac arrest (OHCA) survivors suffer from hypoxic-ischemic brain injury (HIBI), with a high risk of mortality or incidence of poor neurological outcomes without regaining consciousness [1,2]. Based on the “two-hit model,” HIBI can be divided into two phases, namely, primary and secondary injury, which occur during cardiac arrest (CA) and immediately after return of spontaneous circulation (ROSC), respectively [3]. The neuroprotective strategies for managing secondary brain injury, including physiological, pharmacological, or surgical interventions, induce limited improvement of patient outcomes, as the primary brain injury is crucially involved in the possible occurrence of advanced brain injury before applying neuroprotective strategies [4,5]. Studies have reported irreversible brain damage in OHCA survivors through an analysis of the ratio of gray–white matter ratio attenuation (GWR) in brain computed tomography (CT) performed within 24 h of ROSC [6,7,8].

Current international guidelines for the early management of acute ischemic stroke recommend determining whether the primary brain injury is reversible before reperfusion therapy [9]. However, in OHCA survivors, the degree of primary brain injury cannot be ascertained before reperfusion (through ROSC or extracorporeal membrane oxygenation (ECMO)); thus, it is estimated a few hours after reperfusion. Moreover, neuroprotective strategies against secondary brain injury, which involve the modulation of physiological factors (PFs), including arterial carbon dioxide tension (PaCO_2_), arterial oxygen tension (PaO_2_), and mean arterial pressure (MAP), have been uniformly applied in post-cardiac arrest (PCA) care without considering the severity of primary brain injury [10,11,12].

We aimed to assess the associations of PFs (PaCO_2_, PaO_2_, MAP, intracranial pressure (ICP), blood-brain barrier (BBB) status) affecting cerebral perfusion pressure (CPP) with neurological outcomes in OHCA survivors treated with targeted temperature management (TTM). Furthermore, we analyzed the stratifications of those PFs by dividing the patients into total cohort and subgroup analysis, excluding those who had sustained considerable primary brain injury. This was measured by using the high signal intensity (HSI) on diffusion-weighted image (DWI) performed before PCA care [13] because a previous study assessing the usefulness of brain magnetic resonance image (MRI) performed before PCA care showed good performance in detecting already established brain injury after OHCA [14]. We therefore attempted to assess the pure effect of PFs with respect to secondary brain injury, which is targeted for management in PCA care, by avoiding confounding effects from fairly advanced brain injury before PCA care (i.e., the presence of HSI on DWI) that may be irreversible.

## 2. Materials and Methods

### 2.1. Study Setting, Design, and Participants

We performed a retrospective analysis of data prospectively collected from adult comatose OHCA survivors treated with TTM at Chungnam National University Hospital in Daejeon, South Korea, between May 2018 and April 2021. Our hospital is a tertiary-care university hospital with 71 intensive care unit beds, and the annual number of admissions from the emergency department (ED) to the intensive care unit is approximately 1250. Our OHCA registry includes all necessary information for consecutive data from the onset of OHCA to the final outcome. We excluded patients who could not undergo brain MRI before PCA care. The study was conducted according to the guidelines of the Declaration of Helsinki and approved by the Institutional Review Board of Chungnam National University Hospital (CNUH 2021-05-048).

### 2.2. Targeted Temperature Management Protocol

The patients underwent TTM as described in a previous study [15]. Briefly, TTM was performed using ice packs, intravenous cold saline, and TTM devices, i.e., Arctic Sun^®^ and Energy Transfer Pads™ (Medivance, Louisville, CO, USA). A target temperature of 33 °C was maintained for 24 h and monitored using a bladder or esophageal probe. After the TTM maintenance phase, patients were rewarmed at 0.25 °C/h to 37 °C. Midazolam (0.05 mg/kg intravenous bolus, followed by titrated continuous intravenous infusion at 0.05–0.2 mg/kg/h) and cisatracurium or rocuronium (0.15 mg/kg intravenous bolus, followed by infusion up to 0.3 mg/kg/h) were administered for sedation and to control shivering. All patients received standard intensive care following our institutional intensive care unit protocol. Based on the 2015 or 2021 international guidelines for PCA care [16,17], intensive care specialists maintained normal-to-mild hypercapnia (35–45 mmHg), normal-to-moderate hyperoxia (75–150 mmHg), and normal-to-high-normal MAP (65–100 mmHg) by monitoring end-tidal carbon dioxide and frequent arterial blood gas analysis (ABG), as well as providing fluid therapy or continuous infusion of noradrenaline or inotropes, at the clinician’s discretion.

### 2.3. Data Collection and Clinical Variables

We extracted the following data from electronic medical records: demographics, comorbidities, CA characteristics, sequential organ failure assessment score, induction time to 33 °C, time to lumbar catheterization, and time to MRI after ROSC. Moreover, prognostic examinations at baseline and/or 24 h after ROSC, including serum neuron-specific enolase (NSE) values and GWR, were extracted. We extracted ICP data and the quotient albumin ratio (Qa), which reflected BBB disruption [18], at baseline (ICP_base_ and Qa_0_) and 24 h (ICP_24_ and Qa_24_) after ROSC. Those PFs (Qa and ICP) were calculated using cerebrospinal fluid (CSF) samples and measured using a Hermetic TM lumbar accessory kit (Integra Neurosciences, Plainsboro, NJ, USA), manometer (between April 2018 and December 2018), and LiquoGuard^®^ pump system (Möller-Medical, Fulda, Germany) (between January 2019 and December 2020), respectively, as described in our previously published study [19]. Lumbar catheterization was performed with the patient lying in the lateral decubitus position, with the neck, hips, and knees flexed. The lumbar catheter was inserted using aseptically-guided sonography by an expert physician. If the patients were ineligible for lumbar catheter placement (e.g., no consent from the patient’s family; requirement to maintain anticoagulation or antiplatelet therapy after early percutaneous coronary intervention or extracorporeal membrane oxygenation; or severe brain edema in imaging studies, such as obliteration of the basal cisterns, pseudo-subarachnoid hemorrhage, and visible intracranial mass), it was not performed.

Additionally, we analyzed modifiable PFs, including PaO_2_, PaCO_2_, and MAP. To estimate the exposure levels from these PFs at different time periods, we calculated the time-weighted average (TWA) for 6 (TWA_6_) and 24 h (TWA_24_) after ROSC. We multiplied the duration that the PFs remained at a specific value by that value, summed these values, and divided it by the total observation duration. Thus, the equation is as follows:

(1) $$\mathrm{TWA}=\sum_{k=1}^{n}Value\;of\;physiologic\;factors_{k\;}\times Time_{k}/\sum_{k=1}^{n}Time_{k}$$

The results of ABGs before arrival at our ED were excluded from the calculation of TWA in the patients who were transferred to our hospital for PCA care. ABG samples were analyzed using a blood gas analyzer (GEM Premier 3500, Instrumentation Laboratory Company, Bedford, MA, USA) and recorded.

### 2.4. Measurement of HSI in DWI

Since the usefulness of DWI in MRI for prognostication in OHCA survivors has been reported previously [20], we extracted DWI data obtained before PCA care to estimate the primary brain injury before PCA care. Continuous DWI sections (40 per patient) were acquired using a 3-T whole-body scanner (Achieva 3T, Philips Healthcare, Best, The Netherlands). Axial proton density/T2-weighted turbo spin-echo fat-suppressed sequences were used. Scanning parameters were as follows: repetition time, 3000 ms; echo time, 80 ms; slice thickness, 3 mm; and spacing between slices, 4 mm. A standard of b = 1000 s/mm^2^ was used for all DWI. Images were assessed by an expert neuroradiologist blinded to the patients’ information and neurologic outcomes. The MRI was set to positive when there was HSI on DWI, regardless of the volume and location following ischemic injury.

### 2.5. Outcomes

The primary outcome in this study was the stratification of PFs affecting CPP in two groups, i.e., whether there was already established primary brain injury before PCA care or not (HSI and non-HSI groups). Furthermore, to analyze the association between PFs and secondary brain injury after excluding the confounding effect of already established brain injury (primary brain injury), we performed a subgroup analysis, whereby we assessed the association of PFs with neurological outcomes. Using the Glasgow–Pittsburgh Cerebral Performance Category (CPC) scale, neurological outcomes were determined as good (CPC 1 or 2) or poor (CPC 3 to 5). The interviews were conducted by an emergency physician who was well-versed with our protocols and blinded to the patients’ prognosis and clinical data.

### 2.6. Statistical Analysis

Categorical and continuous variables are presented as frequencies with proportions and medians with interquartile ranges (IQR), respectively, because all continuous variables in this study showed a non-normal distribution. Categorical and continuous variables were analyzed using Fisher’s exact test and the Mann‒Whitney U test, respectively. We used the Wilcoxon signed-rank test for within-group pairwise comparisons of TWAs of PaCO_2_, PaO_2_, and MAP, as well as ICP and Qa, over time. Statistical analyses were performed using IBM SPSS Statistics for Windows (version 26.0; IBM, Armonk, NY, USA). Statistical significance was set at *p* < 0.05.

## 3. Results

### 3.1. Characteristics of the Study Population

**Figure 1:**
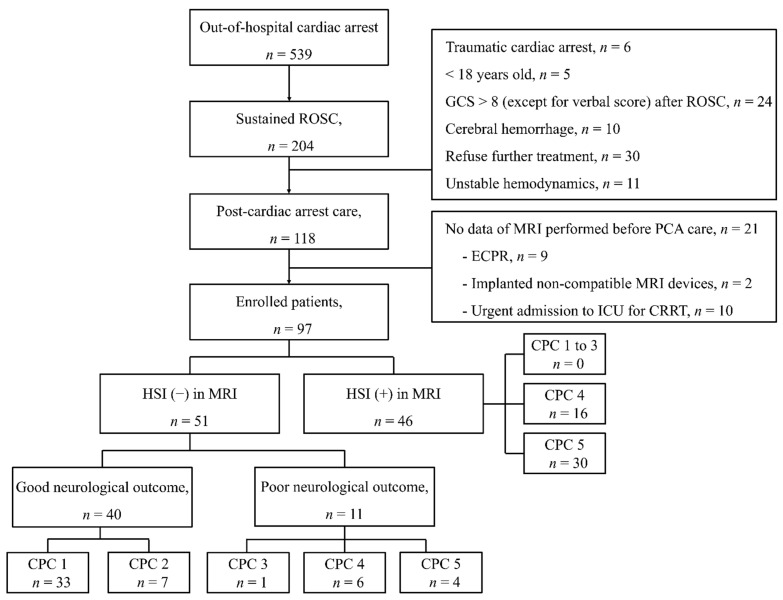
Flow diagram of patient inclusion. Abbreviations: return of spontaneous circulation (ROSC); Glasgow Coma Scale (GCS); magnetic resonance image (MRI); post-cardiac arrest (PCA); extracorporeal cardiopulmonary resuscitation (ECPR); intensive care unit (ICU); continuous renal replace therapy (CRRT); high-signal intensity (HSI); Cerebral Performance Categories (CPC).

The registry included 118 OHCA survivors who underwent TTM; among them, 21 patients were excluded due to lack of MRI data before PCA care for the following reasons: nine received ECMO resuscitation, two had implanted non-compatible MRI devices, and 10 were urgently admitted to the intensive care unit for continuous renal replacement therapy (Figure 1). Finally, we included 97 patients, with 46 patients showing HSI on DWI obtained before PCA care (Figure 1). All patients with HSI on DWI (HSI group) showed poor neurological outcome at 3 months post ROSC. Among the remaining 51 patients with normal DWI findings (non-HSI group), 40 and 11 patients showed good and poor neurological outcomes, respectively (Figure 1).

### 3.2. Characteristics and PFs Associated with HSI on DWI

The HSI group had significantly poorer CA characteristics, including a higher rate of unwitnessed events, non-bystander events, non-shockable rhythm, and non-cardiac etiologies, and longer anoxic time (no and low-flow time) than the non-HSI group (Table 1). Moreover, the HSI group showed significantly higher NSE values and lower GWR after ROSC than the non-HSI group (Table 1).

**Figure 2:**
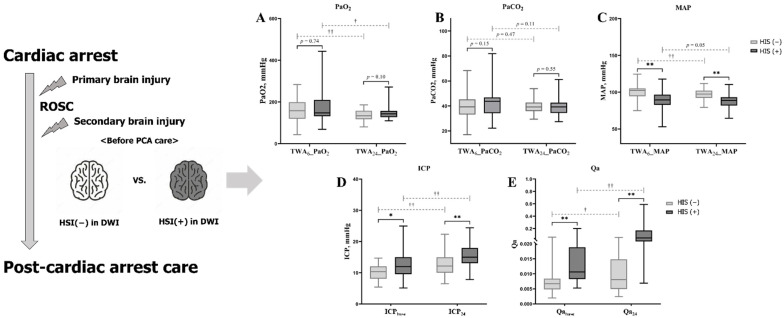
PFs affecting CPP in patients with and without HSI. This analysis was based on DWI obtained before PCA care. Statistical comparison of PFs ((**A**), PaO_2_; (**B**) PaCO_2_; (**C**) MAP; (**D**) ICP; (**E**), Qa) in inter-groups and intra-groups over time. (*) *p* value < 0.05 and (**) *p* value < 0.01, Mann-Whitney U test was performed to comparison in inter-groups. (†) *p* value < 0.05 and (††) *p* value < 0.01, Wilcoxon signed-rank test to pairwise comparison in intra-groups over time. Non-significant *p* values in pairwise comparison between intra-group over time (TWA_6_ or Baseline vs. TWA_24_ or At 24 h): PaCO_2_ in non-HSI and HSI group, *p* = 0.47 and 0.11; and MAP in HSI group, *p* = 0.05 Abbreviations: return of spontaneous circulation (ROSC); post-cardiac arrest (PCA); high-signal intensity (HSI); diffusion-weighted image (DWI); time-weighted average (TWA); arterial oxygen tension (PaO_2_); arterial carbon dioxide tension (PaCO_2_); mean arterial pressure (MAP); intracranial pressure (ICP); albumin quotient (Qa).

The TWA_6_ and TWA_24_ for MAP were significantly lower in the HSI group than in the non-HSI group (Figure 2). Furthermore, PaO_2_ in both groups and MAP in the non-HSI group significantly decreased over time (Figure 2). The ICP and Qa in the HSI group were significantly higher at baseline (ICP, 10.4 mmHg (IQR, 5.4–14.7)) vs. 12.0 mmHg (IQR, 5.2–25.0), *p* = 0.02; Qa, 0.0067 (IQR, 0.0020–0.0613) vs. 0.0107 (IQR, 0.0053–0.2000), *p* < 0.01; (Table 1 and Figure 2) and 24 h (ICP, 12.2 mmHg (IQR, 6.5–22.4)) vs. 15.0 mmHg (IQR, 7.9–24.4), *p* < 0.01; Qa, 0.0081 (IQR, 0.0024–0.0556) vs. 0.0484 (IQR, 0.0069–0.5893), *p* < 0.01; (Table 1 and Figure 2). In the pairwise comparisons of ICP and Qa, both groups showed a significant increase over time (Figure 2).

### 3.3. Characteristics and PFs Associated with Neurological Outcome within the Non-HSI Group

**Figure 3:**
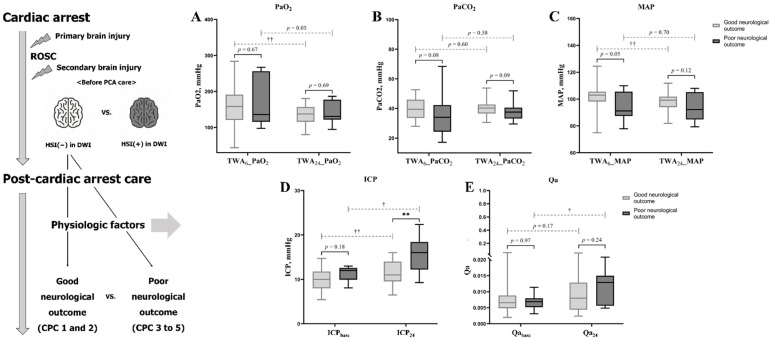
PFs affecting CPP in OHCA survivors with good or poor neurological outcomes. This analysis was conducted in OHCA survivors who either developed a good or poor neurological outcome despite normal findings on DWI obtained before PCA care. Statistical comparison of PFs ((**A**), PaO_2_; (**B**), PaCO_2_; (**C**), MAP; (**D**), ICP; (**E**), Qa) in inter-groups and intra-groups over time. (**) *p* value < 0.01, Mann-Whitney U test was performed to comparison in inter-groups. (†) *p* value < 0.05 and (††) *p* value < 0.01, Wilcoxon signed-rank test to pairwise comparison in intra-groups over time. Non-significant *p* values in pairwise comparison between intra-group over time (TWA_6_ or Baseline vs. TWA_24_ or At 24 h): PaO_2_ in poor neurological outcome groups, *p* = 0.05; PaCO_2_ in good and poor neurological outcome groups, *p* = 0.60 and 0.58; MAP in poor neurological outcome group, *p* = 0.70; and Qa in good neurological outcome group, *p* = 0.17 Abbreviations: return of spontaneous circulation (ROSC); post-cardiac arrest (PCA); high-signal intensity (HSI); diffusion-weighted image (DWI); time-weighted average (TWA); arterial oxygen tension (PaO_2_); arterial carbon dioxide tension (PaCO_2_); mean arterial pressure (MAP); intracranial pressure (ICP); albumin quotient (Qa).

There were no significant differences in CA characteristics, except for the presence of a bystander (34 (85.0%) vs. 6 (54.5%), *p* = 0.04) between patients with good and poor neurological outcomes in the non-HSI group (Table 2). Moreover, serum NSE levels and GWR at baseline and/or 24 h after ROSC did not show a significant inter-subgroup difference (Table 2). There were no inter-subgroup differences in the TWAs of PaO_2_, PaCO_2_, and MAP (*p* > 0.05, Table 2 and Figure 3).

However, the ICP at 24 h after ROSC was significantly higher in patients with poor neurological outcome than in those with good neurological outcome (11.0 mmHg (IQR, 6.5–16.0)) vs. 16.0 mmHg (IQR, 9.3–22.4), *p* < 0.01 (Table 2 and Figure 3). Qa showed a significant increase over time (0.0069 (IQR, 0.0031–0.0114)) to 0.0129 (IQR, 0.0049–0.0294), *p* = 0.04 (Figure 3) in the poor neurological outcomes group, but not in the good outcomes group.

## 4. Discussion

All patients in the HSI group showed significantly poorer CA characteristics, higher ICP, and more severe BBB disruption than those in the non-HSI group. Furthermore, after excluding the confounding effect of primary brain injury, high ICP and BBB disruption at 24 h were significantly associated with poor neurological outcome, which could be attributed to secondary brain injury.

All patients in the HSI group eventually progressed to poor neurological outcomes; in contrast, approximately 22% of patients in the non-HSI group showed poor neurological outcomes. Our findings suggest that it is highly unlikely for CA survivors with HSI on DWI obtained before PCA care to have good neurological outcomes. Moreover, patients with HIBI after CA are likely to have a cerebral penumbra that confers to the risk of additional brain injury, and a consequent secondary brain injury could lead to poor neurological outcomes, regardless of the severity of primary brain injury. Finally, our findings indicate that current strategies for neuroprotection against secondary brain injury after CA could be insufficient in some patients.

In DWI, HSI manifests as cerebral edema of primarily cellular origin, including cellular swelling and subtle changes in tissue-water content [21]. We observed significant intergroup differences in ICP and BBB disruption at the baseline (before PCA care), which became more prominent over time even with standard intensive care for TTM and PFs (PaCO_2_, PaO_2_, and MAP) based on the current international guidelines for PCA care [17]. Our findings suggest that patients with HSI on DWI initially have an established severe brain injury due to both cellular and vasogenic cerebral edema. Additionally, the HSI group showed worse prognostic examination results (NSE and GWR) even at baseline than the non-HSI group. Our findings suggest that HSI on DWI obtained before PCA care is indicative of established severe brain injury sustained by prehospital factors and could reflect irreversible brain injury. Previous studies have demonstrated that diffuse cerebral edema on imaging within 24 h after CA could be indicative of irreversible brain injury, which is consistent with our findings [7,8]. However, visible cerebral edema on imaging performed before PCA care should not necessarily justify therapeutic nihilism, as severe primary brain injury results in poor outcomes by contributing to HIBI; this includes secondary brain injury, rather than a direct effect [22]. Therefore, adequate management of secondary brain injury might provide a significant opportunity in promoting neurological recovery for patients with OHCA.

Several clinical randomized controlled trials (RCTs) have evaluated the modulation of PFs that affect CPP, including PaO_2_, PaCO_2_, and MAP [10,11,12,23], for managing brain injury during PCA care. Despite several attempts to adequately maintain CPP, the modulation of PFs did not significantly improve neurological outcomes in patients with HIBI [10,11,12,24]. The neuroprotective effect of modifying PFs may be concealed by the dominant effect of preexisting primary brain injury on an evolving secondary brain injury during PCA care. This is because the abovementioned RCTs did not consider the confounding effects of primary brain injury on secondary brain injury. However, this explanation remains insufficient because experimental studies on the neuroprotective effect of modifiable PFs under strictly controlled primary brain injury did not show significant neurological recovery [25,26,27]. Furthermore, previous studies have reported heterogeneity among CA survivors in maintaining their own optimal CPP [28], as well as different pathophysiological phenotypes in HIBI according to oxygen transport to the cerebral parenchyma dependent on the presence of lung abnormalities following resuscitation [29,30]. Moreover, current international guidelines recommend that unified physiological targets could be insufficient for the management of CA survivors suffering from secondary brain injury.

In our subgroup analysis performed after excluding the confounding effect of primary brain injury, the poor neurological outcome subgroup showed significantly higher ICP and BBB disruption at 24 h than the good neurological outcome subgroup, despite the non-significant inter-subgroup differences in PaO_2_, PaCO_2_, and MAP that affect CPP. After brain injury, pathological BBB disruption is crucially involved in cerebral edema formation, which increases ICP [31,32]. A recent study reported that individualized intervention for normalizing brain tissue oxygenation and ICP was associated with good neurological outcomes after CA [33]. Furthermore, Naito et al. investigated a cohort of nine patients with cardiac arrest who underwent mild therapeutic hypothermia (34 °C) and reported non-significant differences in ICP between CPC 1 and 2; and CPC 3 and 4, while the patients with CPC 5 showed significantly higher ICP than those of CPC 1–4 [34]. We, therefore, suggest that the normal cut-off value of ICP in cardiac arrest could be different in healthy individuals. This warrants confirmation from a future prospective study with a large sample size. In summary, our findings further confirm that cerebral edema from increased ICP and BBB disruption could be a significant mediator of secondary brain injury [21]. Moreover, improved HIBI management, including individualized CPP optimization via the management of ICP and/or BBB disruption, may allow significant opportunities to improve neurological outcomes in patients with HIBI after OHCA in the future.

This study has several limitations. First, this was a single-center small-scale retrospective study; therefore, we can only report the relationship of CA characteristics and PFs with primary or secondary brain injury; however, we cannot determine a causal relationship. We recommend further large-scale studies to determine the generalizability of this study’s results. Second, we excluded 21 patients who underwent TTM due to their ineligibility for MRI, which could have led to selection bias and limited the generalizability of these results. Given the limited MRI availability, GWR measured using brain CT is widely used to estimate primary brain injury; however, prognostic examinations using brain CT have low sensitivity and inter- or intra-observer reliability [35]. Therefore, the advantages of MRI, including accuracy and reliability, for identifying HSI in DWI could be necessary for excluding primary brain injury sustained before PCA care. Third, it may be possible that brain MRI performed within 6 h from ROSC cannot detect primary brain injuries that develop >6 h after ROSC [36]; furthermore, both the primary and secondary brain injuries affect the HSI on DWI, despite our protocol of performing the imaging as soon as possible after ROSC. Nonetheless, we required the relatively accurate tool to detect an already established brain injury before PCA care for analyses in the total cohort and subgroup. We chose brain MRI because it showed good sensitivity and specificity within a few hours from ROSC to date (AUC 0.80, sensitivity 60.0% (95% CI, 32.3–83.7) with 0% false positive ratio) [14]. Fourth, the ICP and Qa data could not be extracted in 12 patients due to missing data. Specifically, six, five, and one patient could not undergo lumbar catheterization due to the presence of complete basal cistern effacement on brain CT, use of antiplatelet and anticoagulation therapy, and presence of coagulopathy, respectively. We speculate that the six patients with severe cerebral edema could have intracranial hypertension and severe BBB disruption. However, the remaining six patients could have introduced bias due to missing data. Fifth, the gold standard method to measure ICP and Qa is still accepted as external ventricular drain. In particular, there may be bias from the overestimation of CSF albumin. Nonetheless, we suggest that spinal CSF sampling could be helpful for further research as the safety of this technique is comparable to that of external ventricular drain, if the patients are selected appropriately and proper techniques are employed [37], since CSF components except for red-blood cells trend similarly between the cranial and spinal CSF samples with a strong correlation [38]. Furthermore, the sample size calculation could not be performed in this study, because there remains a lack of studies with sufficient sample size for presenting Qa and ICP as the mean ± standard deviation. Future studies with sufficient sample size are required to determine the power of study that is required. Finally, patients with poor neurological outcomes in the non-HSI group showed numerically worse CA characteristics and prehospital factors compared to those with good neurological outcomes. These factors may affect the final neurological outcome at 3 months after ROSC. Notably, the non-significant intergroup differences in the serum NSE levels from baseline to those at 24 h could alleviate the possibility of bias, which results in worse characteristics and prehospital factors of the poor neurological outcome subgroup than in the good neurological outcome subgroup in the non-HSI group.

## 5. Conclusions

There was an association between poor CA characteristics and prehospital factors with HSI on DWI obtained before PCA care. Despite negative findings on DWI before PCA care, OHCA survivors have a cerebral penumbra at risk for an additional brain injury, which could lead to poor neurological outcomes due to a secondary brain injury. Furthermore, ICP and BBB disruption were associated with exacerbated secondary brain injury leading to poor neurological outcomes.

## Figures and Tables

**Table 1:** Baseline demographics, characteristics, and PFs associated between HSI and non-HSI on DWI performed before PCA care.

	Total Number of Patients, *n* = 97	HSI Group *n* = 46	Non-HSI Group, *n* = 51	p-Value
Age, years	57 (16–87)	58 (16–87)	57 (18–83)	0.43
Male	69 (71.1)	32 (69.6)	37 (72.5)	0.51
Pre-existing illnesses				
CAD	7 (7.2)	2 (4.3)	5 (9.8)	0.44
Hypertension	32 (33.0)	17 (37.0)	15 (29.4)	0.52
Diabetes mellitus	29 (29.9)	15 (32.6)	14 (27.5)	0.66
Ischemic stroke	5 (5.2)	3 (6.5)	2 (3.9)	0.67
Pulmonary disease	8 (8.2)	3 (6.5)	5 (9.8)	0.72
Renal disease	14 (14.4)	7 (15.2)	7 (13.7)	1.00
Liver disease	4 (4.1)	1 (2.2)	3 (5.9)	0.62
Cardiac arrest characteristics				
Witnessed	63 (64.9)	26 (56.5)	37 (72.5)	0.01
Bystander CPR	68 (70.1)	28 (60.9)	40 (78.4)	<0.01
Shockable rhythm	27 (27.8)	3 (6.5)	24 (47.1)	<0.01
Cardiac etiology	35 (38.1)	9 (19.6)	26 (51.0)	<0.01
No flow time, min	2.0 (0.0–365.0)	7.0 (0.0–365.0)	5.0 (0.0–330.0)	<0.01
Low flow time, min	19.0 (2.0–58.0)	30.0 (4.0–58.0)	10.0 (2.0–30.0)	<0.01
SOFA score	10 (6–17)	11 (6–16)	9 (6–17)	0.10
GWR before TTM	1.21 (0.97–1.56)	1.17 (0.97–1.35)	1.25 (0.98–1.56)	<0.01
Serum NSE, ng/mL				
at baseline	32.7 (8.5–300.0)	56.0 (13.8–300.0)	25.4 (8.5–68.7)	<0.01
at 24 h from ROSC	35.8 (13.2–300.0)	115.0 (18.2–300.0)	26.5 (13.2–156.7)	<0.01
Induction time to 33 °C, hours	6.0 (2.0–19.0)	5.9 (2.0–19.0)	6.1 (2.6–12.8)	0.92
Time to measure initial ICP, hours	4.6 (1.2–19.5)	4.7 (2.3–19.5)	4.5 (1.2–11.6)	0.55
Time to DWI from ROSC, hours	3.0 (0.9–9.0)	3.0 (0.9–7.8)	3.0 (0.9–9.0)	0.85
PFs				
TWA, mmHg				
Number of performed ABG for 24 h after ROSC	12 (4–19)	12 (4–18)	12 (4–19)	0.76
TWA_6__PaO_2_	149.95 (43.50–442.88)	147.13 (48.48–442.88)	158.23 (43.50–283.78)	0.74
TWA_24__PaO_2_	141.09 (80.20–271.45)	143.40 (109.73–271.45)	133.71 (80.20–186.36)	0.10
TWA_6__PaCO_2_	40.78 (17.11–87.94)	43.82 (22.31–81.94)	39.22 (17.11–68.39)	0.13
TWA_24__PaCO_2_	39.16 (27.52–61.25)	39.21 (27.52–61.25)	39.13 (29.49–53.83)	0.55
TWA_6__MAP	95.93 (52.85–124.50)	89.51 (52.85–117.76)	102.37 (74.97–124.50)	<0.01
TWA_24__MAP	92.96 (64.68–111.89)	88.79 (64.68–110.58)	97.34 (79.41–111.89)	<0.01
ICP, mmHg				
ICP_base_	11.0 (5.2–25.0)	12.0 (5.2–25.0)	10.4 (5.4–14.7)	0.02
ICP_24_	14.0 (6.5–24.4)	15.0 (7.9–24.4)	12.2 (6.5–22.4)	<0.01
Qa				
Qa_base_	0.0083 (0.0020–0.2000)	0.0107 (0.0053–0.2000)	0.0067 (0.0020–0.0613)	<0.01
Qa_24_	0.0161 (0.0024–0.5893)	0.0484 (0.0069–0.5893)	0.0081 (0.0024–0.0556)	<0.01

Data are presented as *n* (%) or median (interquartile range). Abbreviations: diffusion-weighted images (DWI); coronary arterial disease (CAD); cardiopulmonary resuscitation (CPR); sequential organ failure assessment (SOFA); gray–white matter ratio (GWR); targeted temperature management (TTM); neuron-specific enolase (NSE); time-weighted average (TWA) for 6 h (TWA_6_) and 24 h (TWA_24_) from return of spontaneous circulation; partial pressure of oxygen (PaO_2_); partial pressure of carbon dioxide (PaCO_2_); mean arterial pressure (MAP); intracranial pressure (ICP) measured at baseline (ICP_base_) and 24 h (ICP_24_) after return of spontaneous circulation; quotient albumin ratio (Qa) measured at baseline (Qa_base_) and 24 h (Qa_24_) after return of spontaneous circulation.

**Table 2:** Baseline demographics, characteristics, and PFs between good and poor neurological outcomes among patients without HSI on DWI performed before PCA care.

	Total Number of Patients, *n* = 51	Good Neurological Outcome, *n* = 40	Poor Neurological Outcome, *n* = 11	*p*-Value
Age, years	57 (18–83)	58 (18–83)	42 (19–76)	0.34
Male	37 (72.5)	30 (75.0)	7 (63.6)	0.47
Pre-existing illnesses				
CAD	5 (9.8)	5 (12.5)	3 (27.3)	0.57
Hypertension	15 (29.4)	12 (30.0)	3 (27.3)	1.00
Diabetes mellitus	14 (27.5)	11 (27.5)	1 (9.1)	1.00
Ischemic stroke	2 (3.9)	1 (2.5)	1 (9.1)	0.39
Pulmonary disease	5 (9.8)	4 (10.0)	1 (9.1)	1.00
Renal disease	7 (13.7)	7 (17.5)	0	0.32
Liver disease	3 (5.9)	1 (2.5)	2 (18.2)	0.11
Cardiac arrest characteristics				
Witnessed	37 (72.5)	32 (80.0)	5 (45.5)	0.05
Bystander CPR	40 (78.4)	34 (85.0)	6 (54.5)	0.04
Shockable rhythm	24 (47.1)	21 (52.5)	3 (27.3)	0.18
Cardiac etiology	26 (51.0)	22 (55.0)	4 (36.4)	0.32
No flow time, min	5.0 (0.0–330)	0.0 (0.0–33.0)	2.0 (0.0–20.0)	0.36
Low flow time, min	10.0 (2.0–30.0)	10.0 (2.0–30.0)	14.0 (2.0–30.0)	0.44
SOFA score	9 (6–17)	9 (6–17)	9 (7–15)	0.50
GWR before TTM	1.25 (0.98–1.56)	1.25 (1.10–1.56)	1.21 (0.98–1.40)	0.12
Serum NSE, ng/mL				
at baseline	25.4 (8.5–68.7)	25.7 (8.5–54.8)	24.3 (16.1–68.7)	0.99
at 24 h from ROSC	26.5 (13.2–156.7)	26.4 (13.2–85.3)	26.6 (14.6–156.7)	0.97
Induction time to 33 °C, hours	6.1 (2.6–12.8)	5.6 (2.6–12.8)	6.4 (5.5–8.8)	0.10
Time to measure initial ICP, hours	4.5 (1.2–11.6)	4.2 (1.2–11.6)	4.9 (3.0–7.5)	0.30
DWI time from ROSC, hours	3.0 (0.9–9.0)	2.7 (0.9–5.8)	3.7 (1.3–9.0)	0.27
PFs				
TWA, mmHg				
Number of performed ABG for 24 h after ROSC	12 (4–19)	11.5 (4–19)	13 (8–15)	0.08
TWA_6__PaO_2_	158.23 (43.50–283.78)	158.41 (43.50–283.78)	135.75 (97.65–267.25)	0.67
TWA_24__PaO_2_	133.71 (80.20–186.36)	137.31 (80.20–180.39)	130.83 (94.53–186.36)	0.69
TWA_6__PaCO_2_	39.22 (17.11–68.39)	39.48 (27.90–52.74)	34.07 (17.11–68.39)	0.08
TWA_24__PaCO_2_	39.13 (29.49–53.83)	40.65 (30.57–53.83)	37.54 (29.49–51.88)	0.09
TWA_6__MAP	102.37 (74.97–124.50)	102.94 (74.97–124.50)	99.15 (81.97–111.89)	0.05
TWA_24__MAP	97.34 (79.41–111.89)	91.22 (77.94–109.92)	92.20 (79.41–107.96)	0.12
ICP, mmHg				
ICP_base_	10.4 (5.4–14.7)	10.0 (5.4–14.7)	12.0 (8.1–13.0)	0.18
ICP_24_	12.2 (6.5–22.4)	11.0 (6.5–16.0)	16.0 (9.3–22.4)	<0.01
Qa				
Qa_base_	0.0067 (0.0020–0.0613)	0.0066 (0.0020–0.0613)	0.0069 (0.0031–0.0114)	0.97
Qa_24_	0.0081 (0.0024–0.0556)	0.0080 (0.0024–0.0556)	0.0129 (0.0049–0.0294)	0.24

Data are presented as *n* (%) or median (interquartile range). Abbreviations: coronary artery disease (CAD); cardiopulmonary resuscitation (CPR); Sequential Organ Failure Assessment (SOFA); gray-white matter ratio (GWR); targeted temperature management (TTM); neuron-specific enolase (NSE); return of spontaneous circulation (ROSC); intracranial pressure (ICP); diffusion-weighted image (DWI); physiologic factors (PFs); arterial blood gas (ABG); time-weighted image (TWA); arterial oxygen tension (PaO_2_); arterial carbon dioxide tension (PaCO_2_); mean arterial pressure (MAP); albumin quotient (Qa).

## Data Availability

The data presented here are available on request from the corresponding author. The data are not publicly available due to ethical issue.
